# Unveiling the heterogeneity of NKT cells in the liver through single cell RNA sequencing

**DOI:** 10.1038/s41598-020-76659-1

**Published:** 2020-11-10

**Authors:** Hao Shen, Chan Gu, Tao Liang, Haifeng Liu, Fan Guo, Xiaolong Liu

**Affiliations:** 1 State Key Laboratory of Cell Biology, CAS Center for Excellence in Molecular Cell Science, Shanghai Institute of Biochemistry and Cell Biology, Chinese Academy of Sciences, University of Chinese Academy of Sciences, Beijing, China; 2 School of Life Sciences, Hangzhou Institute for Advanced Study, UCAS, Hangzhou, 310024 China; 3 School of Life Science and Technology, ShanghaiTech University, Shanghai, 200031 China; 4 Center for Translational Medicine, Ministry of Education Key Laboratory of Birth Defects and Related Diseases of Women and Children, Department of Obstetrics and Gynecology, West China Second Hospital, Sichuan University, Chengdu, 610041 Sichuan China; 5 Ministry of Education Key Laboratory of Bio-Resource and Eco-Environment, College of Life Sciences, Sichuan University, Chengdu, 610041 Sichuan China

**Keywords:** Immunology, Lymphocytes

## Abstract

CD1d-dependent type I NKT cells, which are activated by lipid antigen, are known to play important roles in innate and adaptive immunity, as are a portion of type II NKT cells. However, the heterogeneity of NKT cells, especially NKT-like cells, remains largely unknown. Here, we report the profiling of NKT (NK1.1^+^CD3e^+^) cells in livers from wild type (WT), Jα18-deficient and CD1d-deficient mice by single-cell RNA sequencing. Unbiased transcriptional clustering revealed distinct cell subsets. The transcriptomic profiles identified the well-known CD1d-dependent NKT cells and defined two CD1d-independent NKT cell subsets. In addition, validation of marker genes revealed the differential organ distribution and landscape of NKT cell subsets during liver tumor progression. More importantly, we found that CD1d-independent Sca-1^−^CD62L^+^ NKT cells showed a strong ability to secrete IFN-γ after costimulation with IL-2, IL-12 and IL-18 in vitro. Collectively, our findings provide a comprehensive characterization of NKT cell heterogeneity and unveil a previously undefined functional NKT cell subset.

## Introduction

Natural killer T (NKT) cells have been increasingly reported to play an important role in controlling innate and adaptive immune responses against cancers, inflammatory disorders and infectious diseases^1^. NKT cells are generally defined as CD1d-dependent natural killer-like T cells. However, previous work also indicated that CD1d-independent NK1.1^+^ cells and other semi-invariant T-cell subsets like MAIT cells are sometimes referred to as NKT cells^2^. Recent work on CD1d-unrestricted NKT cells claimed that the current classification of NKT cells places them into three categories: type I, type II and NKT-like cells^3^. Type I NKT cells, also termed invariant NKT (iNKT) cells, are CD1d-restricted T cells that recognize the glycosphingolipid α-galactosylceramide (α-GalCer) and express an invariant T cell receptor (TCR; variable (V) and joining (J) V_α_14J_α_18 chain and limited TCRβ-chain repertoire)^4,5^. Type II NKT cells are also CD1d-restricted T cells with diverse usage of TCRα and β-chains and are reactive to diverse lipid antigens derived from self or microbes^6^. Unlike iNKT cells, only a portion of type II NKT cells have been studied due to the discovery of specific antigens, such as sulfatide^7^. NKT-like cells are CD1d independent^2^ and include all the other NKT cells. For example, the activated CD8^+^ T cells derived CD8^+^NK1.1^+^ T cells and MAIT cells. The activated CD8^+^ T cells derived CD8^+^NK1.1^+^ T cells acquired NK1.1 expression with no expression of Ly49 receptors (Ly-49C/I and Ly-49G2) in mice liver after allogeneic hematopoietic cell transplantation^8^. MAIT cells, which was identified by Shimamura et.al by using CD1d-deficient mice^9^, are depleted in Jα18-deficient mice^10^.

The majority of the literature now has defined NKT cells as a CD1d-dependent cell type^11^. Both type I and type II NKT cells have been shown to play an important role in the regulation of immune responses in cancer and other diseases^1^. The combined use of Jα18-deficient mice that lack type I NKT cells and CD1d-deficient mice that lack type I and type II NKT cells is useful for studying the biological functions of type I and type II NKT cells in vivo^2^. In general, type I and type II NKT cells play opposing roles in the immune regulation of tumor immunity, which concludes that type I NKT cells protect against cancer, while type II NKT cells suppress tumor immunity^1^. However, some reports suggest a suppressive role of type I NKT cells^12,13^ and an antitumor role of type II NKT cells in tumor immunity^14^, which makes the functional relationship of type I and type II NKT cells conflicting in cancer. Moreover, the definition of subsets of CD1d-dependent NKT cells still mainly relies on finding specific antigens, and there is no known antigen that can stimulate all type II NKT cells uniformly^1^. Additionally, CD1d-independent NKT-like cells are more heterogeneous and less thoroughly characterized than CD1d-dependent NKT cells, which makes it more difficult to study their biological function precisely. To date, the classification of NKT cells remains unclear, and the function of NKT cells mainly relies on the discovery of specific antigens for labeling and activating these cells.

As there is no specific marker that can uniformly isolate all CD1d-dependet natural killer-like T cells. In order to investigate the heterogeneity of NKT cells, we performed scRNA-seq on NKT (NK1.1^+^CD3e^+^) cells in livers from WT, Jα18-deficient and CD1d-deficient mice. Unbiased clustering of the cellular transcriptomes grouped cells into 4 distinct clusters, and transcriptional profiles for each subset were identified. By comparison of the scRNA-seq data of liver NKT cells from WT mice with those of liver NKT cells from Jα18-deficient and CD1d-deficient mice, we identified type I and type II NKT cells and divided the CD1d-independent NKT cells into two major subsets. Additionally, we used two cell surface markers to separate the NKT cell subsets and determine their distribution in different organs at steady state or in the liver during liver tumor development. Moreover, we found that CD1d-independent Sca-1^−^CD62L^+^ NKT cells show a specific ability to secrete IFN-γ after costimulation with IL-12, IL-18 and IL-2 in vitro. Taken together, our research reveals the landscape of NKT cells in the liver and redefines the NKT cell subsets.

## Results

### Overview of NKT cell subsets identified by single-cell RNA sequencing in the mouse liver

**Figure 1:**
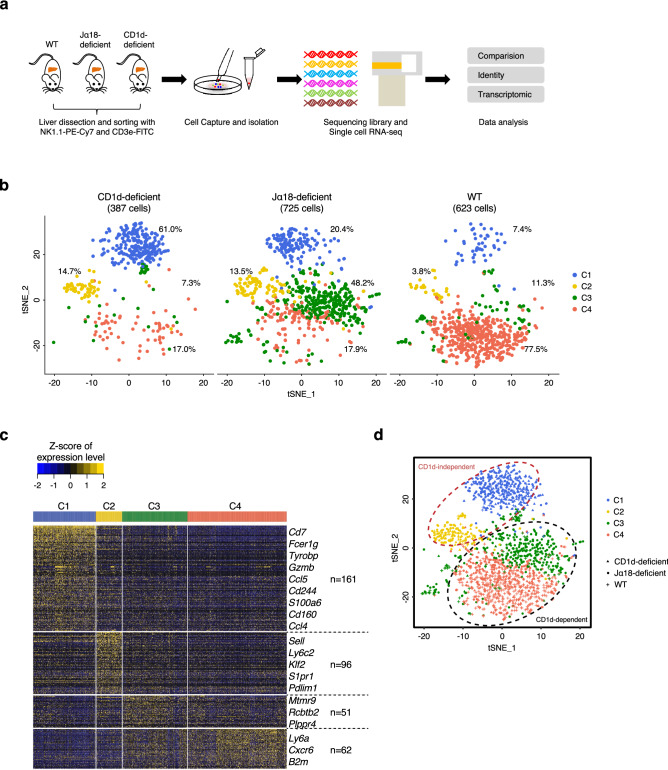
Unbiased single-cell RNA-seq analysis reveals distinct NKT cell subsets in the mouse liver. (**a**) Workflow describes the overview of the single-cell RNA-seq experimental design. (**b**) t-SNE analysis of NKT cells from WT mice (623) or Jα18-deficient mice (725) and CD1d-deficient mice (387). The percentage of cell population was indicated. (**c**) Analysis of the differentially expressed genes among single NKT cells. (**d**) t-SNE analysis of NKT cells from WT mice, Jα18-deficient mice and CD1d-deficient mice, annotated by cell-type identity.

To investigate the heterogeneity of NKT cells, we chose NKT cells from the liver, the peripheral organ with the highest frequency of NKT cells in mice, for single-cell RNA sequencing. A single-cell suspension was prepared from the dissociated liver, and NKT cells were enriched by cell sorting (FACS) of NK1.1^+^CD3e^+^ cells and subjected to scRNA-seq (Fig. 1a). After quality control, 623 cells from the liver in WT mice, 725 cells from the liver in Jα18-deficient mice and 387 cells from the liver in CD1d-deficient mice were used in the downstream analysis. On average, 62,895 UMIs and 2,231 genes were detected in these individual cells (Supplementary Fig. 1). Using t-SNE analysis, 4 distinct clusters of NKT cells were well segregated (Fig. 1b). Cluster 1 (C1) accounted for 7.4% of the cells in WT mice, 20.4% of the cells in Jα18-deficient mice, and 61.0% of the cells in CD1d-deficient mice; Cluster 2 (C2) accounted for 3.8% of the cells in WT mice, 13.5% of the cells in Jα18-deficient mice, and 14.7% of the cells in CD1d-deficient mice; Cluster 3 (C3) accounted for 11.3% of the cells in WT mice, 48.2% of the cells in Jα18-deficient mice, and 7.3% of the cells in CD1d-deficient mice; and Cluster 4 (C4) accounted for 77.5% of the cells in WT mice, 17.9% of the cells in Jα18-deficient mice, and 17.0% of the cells in CD1d-deficient mice. Heatmaps of the normalized NKT cell profiles showed the normalized expression of the top variable genes in each NKT cell subset (Fig. 1c). By comparison of the clustering pattern of NKT cells from WT mice with those of NKT cells from Jα18-deficient and CD1d-deficient mice, the NKT cells were divided into 4 distinct subsets, which we temporarily termed C1, C2, C3 and C4. We identified well-known CD1d-dependent NKT cells (type I and type II NKT cells) and two distinct subsets of CD1d-independent NKT cells (NKT-like cells) (Fig. 1d). C1 and C2 represented two major subsets of CD1d-independent NKT cells. C3 and C4 represented CD1d-dependent type II NKT cells and type I NKT cells. Altogether, these unbiased analyses reveal the diversity of NKT cells in the mouse liver and reclassify the NKT cells into 4 distinct subsets without the need for specific antigens.

### Transcriptional features of distinct NKT cell subsets

**Figure 2:**
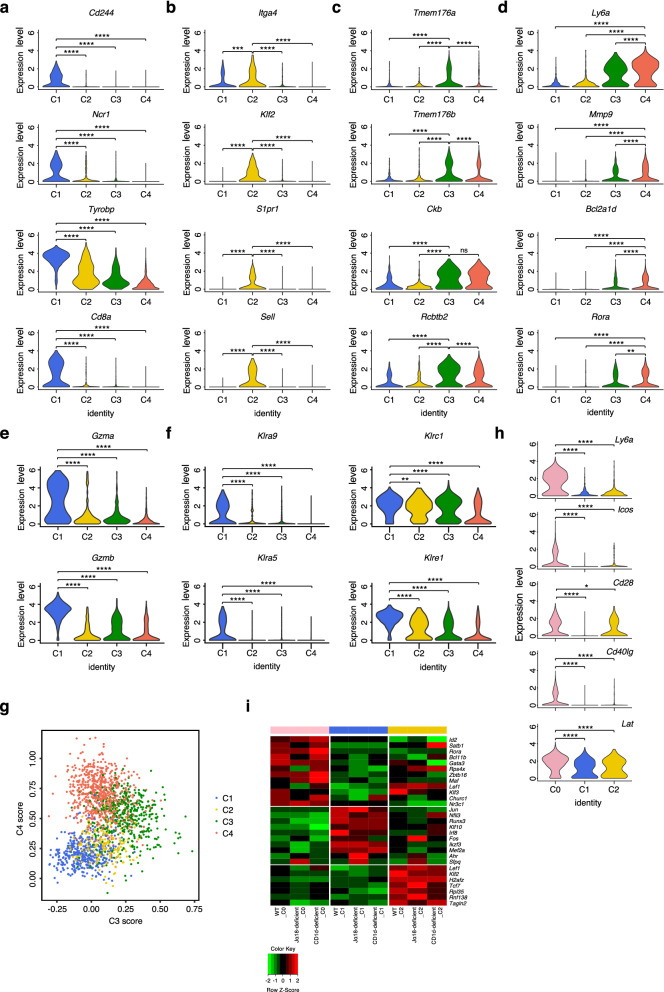
Molecular feature of each NKT cells subsets. (**a–d**) Violin plots showing the differential expression genes of each clusters. (**e**) Violin plots showing the expression of *Gzma* and *Gzmb* across all clusters. (**f**) Violin plots showing the expression of *Klra9*, *Klra5*, *Klrc1* and *Klre1* across all clusters. (**g**) Similarity score across all clusters. (**h**) Violin plots showing the differential expression genes of Cluster 0. (**i**) Heatmaps of detectable transcription factors in NKT cells subsets. Gene expression in each cluster was calculated from the combination of all liver samples from WT, Jα18-deficient and CD1d-deficient mice, unless otherwise indicated. P-values were defined by the Student’s t-test. *P < 0.05; **P < 0.01; ***P < 0.001; ****P < 0.0001 by Student’s t-test; N.S.: no significance.

Next, we sought to explore the transcriptional features of different NKT cell subsets. We identified more than 100 genes that were specifically expressed in the CD1d-independent NKT cell subset C1 via single-cell RNA-seq. Interestingly, many genes were related to the receptors of NK cells, including NK cell receptor 2B4 (*Cd244*), NKp46 (*Ncr1*), TYPO protein tyrosine kinase-binding protein (*Tyrobp*) and some of the killer cell lectin-like receptor (*Klr*) genes (Fig. 2a,f). Additionally, we found that some genes were related to the cytotoxicity pathway, including granzymes (*Gzma* and *Gzmb*) (Fig. 2e). We also found that CD8α (*Cd8α*) was expressed at significantly higher levels in C1 than in other NKT cell subsets, which was consistent with previous studies of cytotoxicity and granules in CD8^+^ NKT cells^15,16^. We also identified approximately 100 genes that were specifically expressed in the CD1d-independent NKT cell subset C2 via single-cell RNA-seq (Fig. 2b). Unlike those enriched in C1, the genes enriched in C2 were related to cell adhesion and cell migration. The gene encoding α4 integrin (*Itga4*), which has been reported to be essential for T cell trafficking and adhesion^17^, was enriched in C2. In addition, the expression levels of *Klf2*, *S1pr1* and *Sell* were significantly higher in C2 than in the other subsets. Previous studies have reported that Krueppel-like factor 2 (*Klf2*) directly promotes naïve T cell recirculation through regulation of the expression of sphingosine 1-phosphate receptor 1 (*S1pr1*) and L-selectin (*Sell*)^18–20^. Therefore, the CD1d-independent NKT cell subset C2 might not be tissue-resident cell types and might participate in immune surveillance and host defense. For CD1d-dependent NKT cells, we identified genes expressed specifically in C3 and C4 cells (Fig. 2c,d). Unlike the genes specifically expressed in C1 or C2, the genes enriched in C3 and C4 showed a similar pattern with each other. Analysis of the similarity of NKT cell subsets showed that C3 and C4 had significantly higher similarity scores than C1 and C2 (Fig. 2g). However, previous studies on NKT cells regarded type I and type II NKT cells as two different NKT subsets with opposite functions^1^. Our data indicated that C3 and C4 might be considered as a single cluster on the basis of detailed analysis at the transcriptional level. To better analyze transcriptionally different subsets of NKT cells, we combined CD1d-dependent NKT cells (C3 and C4) into a single subset called C0. The genes enriched in C0 were related to T cell activation, including *Ly6a*, *Icos*, *Cd28* and *Cd40lg* (Fig. 2h). Compared to other subsets, C0 showed significantly higher expression of *Lat*, an important component of both TCR signaling in T cell development and function and FCGR3-mediated signaling in NK cells^21,22^. In addition, analysis of the expression of transcription factors showed distinct regulation of transcription in NKT cell subsets (Fig. 2i). Notably, we analyzed the transcriptional pattern of genes encoded proteins like CD8α, Ly-49C/I and Ly-49G2 in our classified CD1d-independent populations C1 and C2 (Fig. 2a; Supplementary Fig. 2a) and found that the transcriptional pattern was not similar to the activated CD8^+^ T cells derived CD8^+^NK1.1^+^ T cells (CD8α^+^Ly-49C/I^−^Ly-49G2^−^)^8^, which indicated that both C1 and C2 are not the activated CD8^+^ T cells derived CD8^+^NK1.1^+^ T cells. We also compared the transcriptional profiles of C1 and C2 in wild-type, Jα18-deficient (lack MAIT cells^10^) and CD1d-deficient mice and found that the transcriptional profiles of these two populations were comparable in different mouse models (Fig. 1d), which indicated that MAIT cells are not the major population of either C1 or C2. These results are consistent with previous works that activated CD8^+^ T cells derived CD8^+^NK1.1^+^ T cells expanded only during pathological state but not in steady state^8^ and MAIT cells were generally low in frequency in laboratory mice^23^. Taken together, these results show that NKT cells could be divided into 3 transcriptionally distinct subsets with two major subsets of CD1d-independent NKT cells (termed C1 and C2 below) and a combined subset of CD1d-dependent NKT cells (termed C0), which included C3 and C4.

### Properties of immune regulation among distinct NKT cell subsets

**Figure 3:**
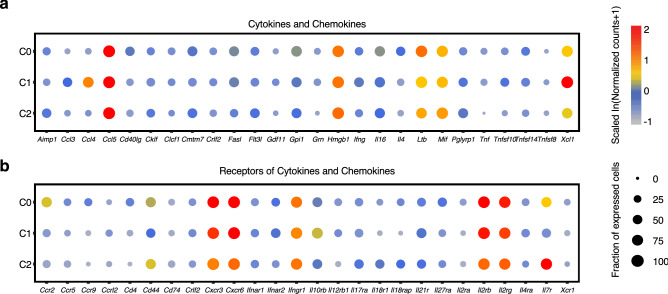
NKT cell subsets showed differential properties in immune regulation. (**a**,**b**) Expression of detectable cytokines, chemokines and their receptors (x axis) in mouse liver NKT cells subsets (y axis). Dot size represents the percentage of cells where the gene is detected. Color indicates the mean expression in a subset relative to other subsets. Gene expression in each cluster was calculated from the combination of all liver samples from WT, Jα18-deficient and CD1d-deficient mice.

To explore whether these transcriptionally distinct NKT cell subsets show different immune regulation properties, we first examined the expression of cytokines and chemokines in the defined clusters (Fig. 3a). C0 had significantly higher expression of *Il4* than the other subsets, which was consistent with the findings that both type I NKT cells and a portion of type II NKT cells can secrete IL-4 upon stimulation^14,24^. C0 also had significantly higher expression of *Cd40lg*, which acts as a costimulatory molecule that participates in T cell activation^25^. For C1, chemokines with inflammatory properties, such as *Ccl3* and *Ccl4*, were expressed at significantly higher levels in C1 than in the other subsets. The expression of *Xcl1*, which plays an important role in the dendritic cell-mediated cytotoxic immune response^26^, was significantly higher in C1 than in the other subsets. Unlike C0 or C1, C2 showed no specifically expressed cytokines or chemokines. In addition, all of the NKT cell subsets had high expression of *Ccl5*, which regulates the recruitment of T cells and neutrophils and has been reported to participate in the antitumor activity of iNKT cells^27^. Other immune regulators, such as *Hmgb1*, *Ltb* and *Mif*, which have not been thoroughly studied in NKT cells, were also enriched in all the NKT cell subsets. Regarding the differential expression of receptors of cytokines and chemokines (Fig. 3b), all the subsets had high expression of *Cxcr3*, which was induced following T cell activation in naïve T cells and reported to be highly expressed in iNKT cells^28,29^. The chemokine receptor *Cxcr6*, a receptor known to be required for the localization of iNKT cells in the liver^30^, was expressed at significantly higher levels in C0 and C1 than in C2. The components of the IL-2 receptor, *Il2rb* and *Il2rg*, were highly expressed in all subsets. In addition, C0 had significantly higher expression of *Ccr2*, which has been reported to promote the differentiation of T cells into T-helper 17 cells (Th17)^31^. Moreover, C2 had significantly higher expression of *Il7r*, which is related to memory signatures^32^, similar to other enriched genes, such as *Sell* and *Tcf7* (Fig. 2b,i), in C2 than in other NKT cell subsets. C2 also had significantly higher expression of *Il18r1* and *Il18rap*, major components of the IL-18 receptor complex that mediate IL-18 signals^33^. Collectively, the NKT cell subsets expressed different molecules involved in multiple biological processes, which might indicate their differential tissue localization and immune regulation.

### Distribution of NKT cell subsets at steady state or during liver tumor development

**Figure 4:**
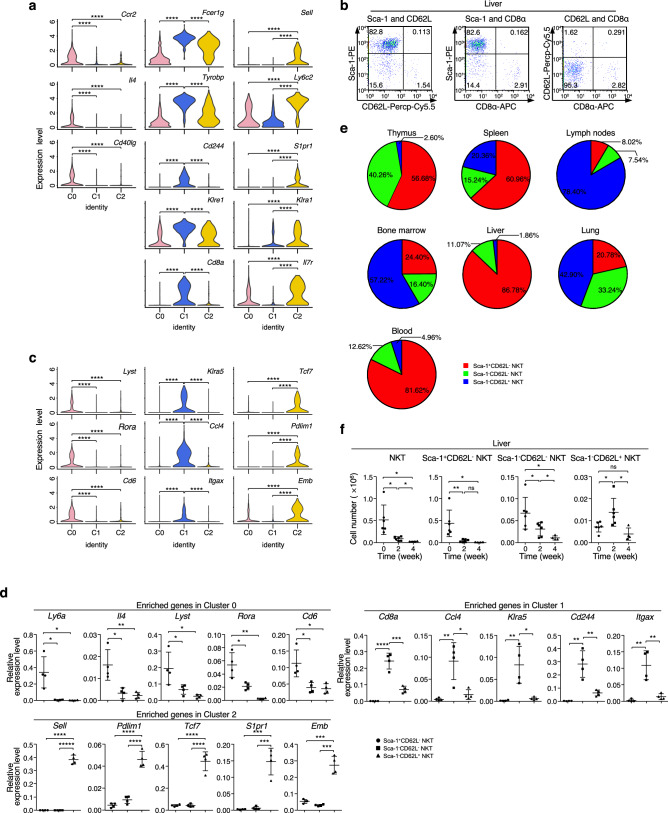
Validation of marker genes and NKT cell subsets distribution during steady or pathological state. (**a**) Violin plots showing the expression of candidate marker genes across all clusters. (**b**) Flow cytometric analysis of the expression of Sca-1, CD62L and CD8α in liver NKT cells from WT mice. (**c**) Violin plots showing the expression of significantly expressed genes across all clusters. (**d**) Quantitative RT-PCR analysis of the mRNA level of significantly expressed genes across all clusters in liver NKT cell subsets as sorted with Sca-1 and CD62L by flow cytometry from WT mice (All genes, n = 4). All the expression levels were normalized to the expression of *Gapdh*. (**e**) Pie charts showing the frequency of NKT cell subsets in NKT cells in different organs from WT mice (n = 5). (**f**) The cell number of NKT cell subsets in the liver from WT mice untreated or treated at the indicated time during liver tumor progression (Untreated, n = 6; 2 weeks, n = 6; 4 weeks, n = 4). For (**a**) and (**c**), gene expression in each cluster was calculated from the combination of all liver samples from WT, Jα18-deficient and CD1d-deficient mice, unless otherwise indicated. P-values were defined by the Student’s t-test. For (**b**) and (**d**–**f**), the data are presented as the mean ± s.d and are representative of or combined from at least three independent experiments, unless otherwise indicated. For all panels: *P < 0.05; **P < 0.01; ***P < 0.001; ****P < 0.0001 by Student’s *t*-test; N.S.: no significance.

No previous work has reported the clustering of NKT cell subsets as described above. To better examine the distribution and function of these NKT cell subsets in vivo, we first analyzed cell membrane proteins as cell-surface markers at the mRNA level. Single-cell data analysis showed specific markers in each cluster (Figs. 2h, 4a). Among these candidate surface proteins, we chose *Ly6a* (protein name: Sca-1), *Sell* (protein name: CD62L) and *Cd8α* (protein name: CD8α) as candidate markers to separate the NKT cells into subsets that represent the subsets (C0, C1 and C2) identified in single-cell data analysis. To explore this possibility, we first analyzed the expression of Sca-1, CD8α and CD62L via FACS analysis (Fig. 4b). The FACS data showed a clear pattern with similar frequencies for the 3 subsets, with C0 corresponding to Sca-1^+^CD62L^−^ NKT cells, C1 corresponding to Sca-1^−^CD62L^−^ NKT cells and C2 corresponding to Sca-1^−^CD62L^+^ NKT cells; the other two patterns were not observed.

To validate the marker genes, we listed the differentially expressed genes in each cluster (Fig. 4c). By cell sorting with Sca-1 and CD62L as cell-surface markers, we confirmed some identified genes (Figs. 2h, 4a,c) at the mRNA level by using real-time PCR (qPCR) analysis (Fig. 4d). We also validated some enriched genes at the protein level (Supplementary Fig. 2b). Additionally, we examined the distribution of NKT cell subsets in different organs (Fig. 4e; Supplementary Fig. 3a). We found that the majority of NKT cells in the liver and blood are CD1d-dependent NKT cells, which was consistent with the finding that iNKT cells were dominant in the liver^34^. However, the frequency of Sca-1^−^CD62L^+^ NKT cells increased in the spleen, a peripheral lymphoid organ that has been well studied in the context of iNKT cells. For organs such as the lung, bone marrow and lymph nodes, at least 40% of NKT cells were Sca-1^−^CD62L^+^ NKT cells. To determine whether the distribution of NKT cell subsets is altered in the pathologic state, we established a liver tumor model via hydrodynamic injection^35^. We found that the cell number of total NKT cells was significantly decreased during tumor progression (Fig. 4f). In detail, the cell number of Sca-1^+^CD62L^−^ NKT cells was significantly decreased in the early stage (two weeks). The cell number of Sca-1^−^CD62L^−^ NKT cells was comparable in the early stage (two weeks) and at steady state and significantly decreased in the late stage (four weeks). Intriguingly, the cell number of Sca-1^−^CD62L^+^ NKT cells was significantly increased in the early stage (two weeks) and decreased in the late stage (four weeks), which indicated that Sca-1^−^CD62L^+^ NKT cells might participate in the immune regulation of tumor progression in the early stage. Taken together, we identified two cell-surface markers as marker genes to separate the NKT cell subsets at the protein level and revealed the landscape of NKT cell distribution at steady state and during liver tumor development.

### Unique function of CD1d-independent Sca-1^−^CD62L^+^ NKT cells in vitro

**Figure 5:**
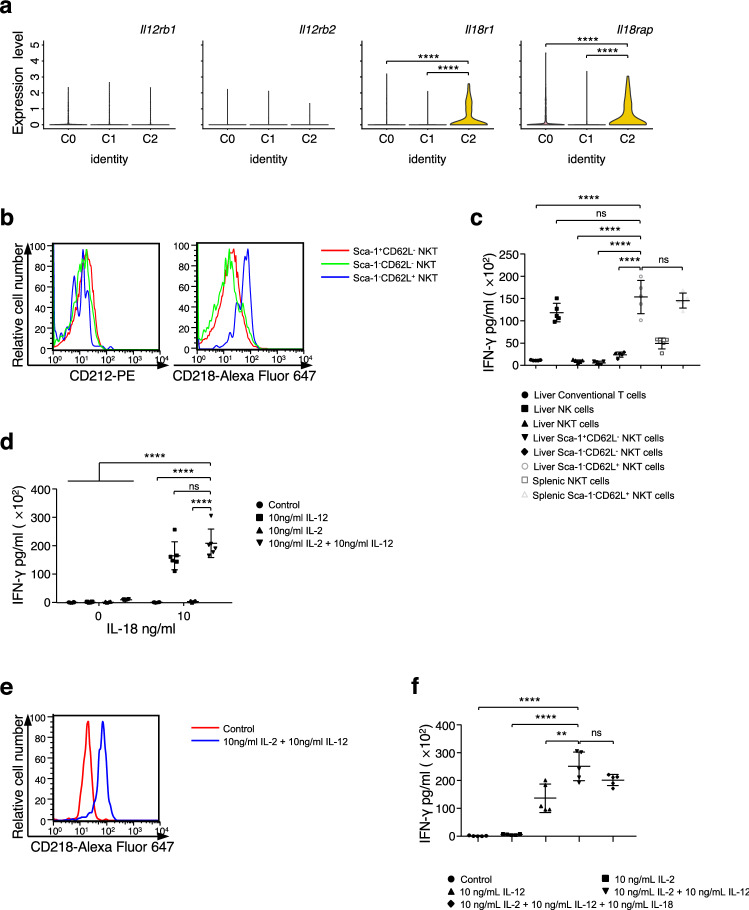
Specific IFN-γ response of CD1d-independent Sca-1^−^CD62L^+^ NKT cells in vitro. (**a**) Violin plots showing the expression of *Il12rb1*, *Il12rb2*, *Il18r1* and *Il18rap* across all clusters. (**b**) Representative histograms of the expression of CD212 and CD218 in NKT cell subsets in the liver from WT mice. (**c**) After cell sorting of distinct cell types from the liver and spleen, 3000 cells of each cell type were treated with 10 ng/mL IL-2, 10 ng/mL IL-12 and 10 ng/mL IL-18 for 48 h. ELISA was performed to measure IFN-γ titers in the supernatant of indicated cell types (n = 5). (**d**) After cell sorting of Sca-1^−^CD62L^+^ NKT cells from the spleen, 3000 cells were treated with indicated conditions for 48 h. ELISA was performed to measure IFN-γ titers in the supernatant (n = 6). (**e**) Representative histograms of the expression of CD218 in sorted Sca-1^−^CD62L^+^ NKT cells untreated or treated with 10 ng/mL IL-2 and 10 ng/mL IL-12 for 24 h. (**f**) After cell sorting of Sca-1^−^CD62L^+^ NKT cells from the spleen, 3000 cells were pretreated with indicated conditions for 24 h and then add the IL-18 to a final concentration of 10 ng/mL for another 48 h. ELISA was performed to measure IFN-γ titers in the supernatant (n = 5). For (**a**), gene expression in each cluster was calculated from the combination of all liver samples from WT, Jα18-deficient and CD1d-deficient mice, unless otherwise indicated. P-values were defined by the Student’s t-test. For (**b**–**f**), the data are presented as the mean ± s.d and are representative of or combined from at least three independent experiments, unless otherwise indicated. For all panels: **P < 0.01; ****P < 0.0001 by Student’s *t*-test; N.S.: no significance.

Due to the specific alterations in the cell number of Sca-1^−^CD62L^+^ NKT cells during liver tumor progression, we explored the unique function of Sca-1^−^CD62L^+^ NKT cells. Our data above has demonstrated that the expression of components of the IL-18 receptor complex was significantly higher in Sca-1^−^CD62L^+^ NKT cells than in other NKT cell subsets. IL-18, an IFN-γ-inducing factor with antitumor activity in murine tumor models, has been reported to enhance the proliferation, cytotoxicity and IFN-γ secretion of both NK and T cells^36–40^. IL-12 and IL-18 are known to induce IFN-γ production in Th1 cells and NK cells^41^. We first analyzed the IL-12 receptor and IL-18 receptor in NKT cell subsets at the single-cell RNA level (Fig. 5a). The expression of *Il12rb1* and *Il12rb2* was virtually undetectable among NKT cell subsets. However, the expression of *Il18rb1* and *Il18rap* was significantly higher in C2 than in the other subsets. These results were further confirmed at the protein level by flow cytometry (Fig. 5b). Thus, we presumed that Sca-1^−^CD62L^+^ NKT cells might have a strong IFN-γ response upon stimulation with the combination of IL-12 and IL-18.

To determine the IFN-γ secretion ability of NKT cell subsets, we sorted conventional T cells, NK cells, total NKT cells and NKT cell subsets from the liver and total NKT cells and Sca-1^−^CD62L^+^ NKT cells from spleen and then stimulated them with IL-12/IL-18 plus IL-2 (a cytokine known to promote the proliferation of both T and NK cells). The production of IFN-γ was analyzed after stimulation for 48 h. As expected, Sca-1^−^CD62L^+^ NKT cells showed significantly higher secretion of IFN-γ than the other NKT cell subsets and T cells and slightly higher secretion of IFN-γ than NK cells (Fig. 5c). Moreover, Sca-1^−^CD62L^+^ NKT cells from the spleen showed comparable IFN-γ production to with those from the liver in vitro, which suggested that Sca-1^−^CD62L^+^ NKT cells might serve as immune regulators that travel among different organs. However, whether the transcriptome of Sca-1^−^CD62L^+^ NKT cells is altered in different organs needs to be explored in the furture. Additionally, we were able to use Sca-1^−^CD62L^+^ NKT cells from the spleen, which contained more total cells than the liver in each mouse (Supplementary Fig. 3b), for a detailed study.

To further analyze the effect of the cytokines of IL-2, IL-12 and IL-18 on the IFN-γ response of Sca-1^−^CD62L^+^ NKT cells, we first examined the need for IL-2, IL-12 and IL-18 (Fig. 5d). Stimulation with IL-12 or IL-18 alone could not induce a strong IFN-γ response in Sca-1^−^CD62L^+^ NKT cells. The cytokine IL-2 slightly enhanced the IFN-γ response of Sca-1^−^CD62L^+^ NKT cells. Previous studies have reported that the function of IL-18 is the induction of IFN-γ production^36^. Therefore, we assumed that Sca-1^−^CD62L^+^ NKT cells might take up IL-12 and IL-2 and upregulate the expression of the IL-18 receptor to amplify the IFN-γ response, which was reported in T cells^41^. As expected, treatment of Sca-1^−^CD62L^+^ NKT cells with IL-2 and IL-12 alone for 24 h upregulated the expression of IL-18R1 (Fig. 5e), and stimulation of Sca-1^−^CD62L^+^ NKT cells via pretreatment with IL-2 and IL-12 for 24 h slightly enhanced IFN-γ production (Fig. 5f). Overall, we concluded that Sca-1^−^CD62L^+^ NKT cells undergo a specific IFN-γ response after stimulation with the combination of IL-2, IL-12 and IL-18.

## Discussion

In this study, benefiting from the advantages of single-cell RNA-seq, we showed the heterogeneity of NKT cells from the mouse liver regardless of antigen specificity. We identified the well-known CD1d-dependent NKT cells (both type I and type II NKT cells) and divided the CD1d-independent NKT cells into two major subsets. Transcriptome profiling revealed distinct characteristics of each subset in different biological processes and immune regulation. In addition, the validation and usage of Sca-1 and CD62L as cell surface markers helped us to explore the distribution of NKT cell subsets in different organs and during liver tumor development. Moreover, we found a unique IFN-γ response among Sca-1^−^CD62L^+^ CD1d-independent NKT cells, while the other NKT cell subsets had no response. Overall, our work provides a comprehensive gene expression map of divergent subsets of NKT cells in the mouse liver, which will facilitate the understanding of the heterogeneity of NKT cells and the studies of CD1d-independent NKT cells.

Due to the discovery of specific antigens, such as α-GalCer and sulfatide, a large number of studies of NKT cells have focused on the development and function of CD1d-dependent NKT cells. To date, the defined CD1d-dependent NKT cells have included type I NKT cells (α-GalCer-reactivity), one subset of type II NKT cells (sulfatide-reactivity) and another subset of type II NKT cells (β-GlcCer-reactivity)^4,7,14^. And due to the lack of a specific marker to isolate all subsets of type II NKT cells, the transcriptional profile of type II NKT cells remains obscure, so does the CD1d-independent NKT cells. The combined use of Jα18-deficient mice that lack type I NKT cells and CD1d-deficient mice that lack type I and type II NKT cells has been widely used to study the biological functions of type I and type II NKT cells in vivo^2^. By comparison of data from WT, Jα18-deficient and CD1d-deficient mice (Fig. 1d), our work provided the transcriptional profile of the well-known type I and type II NKT cells and two distinct CD1d-independent NKT cell subsets, which has not been described except the type I NKT cells^42,43^. Although recent works have pointed out some limitations of these mouse models. The original Jα18-deficient mice has multiple Jα elements deleted that constricted the T cell receptor repertoire^44^. Similarly, the presence or absence of the CD1d2 in CD1d-deficient mice might alter the pool of the CD1d-independent NKT cells, which was not deleted in our CD1d-deficient mice^45^. Besides, the absence of type I and type II NKT cells might impact the cytokine milieu and thus could potentially affect the transcriptional profile of the remaining CD1d-independent NKT cells. In spite of these, the combination of the single-cell RNA sequencing data in our work from WT, Jα18-deficient and CD1d-deficient mice showed similar patterns (Fig. 1d), which indicated that the transcriptional profiles of NKT cell subsets were comparable in different mouse models.

It is difficult to fully understand CD1d-dependent NKT cells due to the diversity of TCR usage among type II NKT cells^6^. Due to our sorting strategy which was mainly focused on NK1.1^+^ NKT cells, we excluded a minority of NK1.1^−^ hepatic type I NKT cells that comprised about 15% of total type I NKT cells in our system (Supplementary Fig. 3c,d). Despite of this, we were able to analyzed the transcriptomes of NK1.1^+^ type I NKT cells and type II NKT cells and found that they are similar to each other. This unexpected finding indicated that the understanding of CD1d-dependent NKT cells in the liver might not be so comprehensive, at least at the mRNA level. Many studies on NKT cell function in tumors have concluded that type I NKT cells have antitumor ability and that the roles of type II NKT cells are mostly associated with tumor suppression^1,46–50^. However, Jie Zhao et al. showed that a subset of type II NKT cells also contributed to the antitumor effect of CpG in the B16 melanoma model^14^. The functional relationship between type I and type II NKT cells is still controversial. Our single-cell RNA-seq data on type I and type II NKT cells might provide an opportunity to determine the similarities and differences between these two types of CD1d-dependent NKT cells at the transcriptional level. In addition, type II NKT cells are more widespread than type I NKT cells in humans, which differs from the predominance of type I NKT cells in mice^6,51^. Therefore, much effort needs to be made to understand CD1d-dependent NKT cells more comprehensively in the future, which requires more research on NKT cells from both mice and humans with single-cell resolution.

In contrast to the subsets of CD1d-dependent NKT cells, NKT-like cells comprised the rest of the NKT cells in a CD1d-independent manner^2^. They are now the most heterogeneous subset, with no standard classification, which makes the study of CD1d-independent NKT cells more difficult and controversial. In our study, we found that CD1d-independent NKT cells can be divided into two major subsets. The transcriptome of one subset is highly related to the cytotoxicity pathway, with abundant granules at the mRNA level and higher expression of CD8α, which was consistent with previous works on CD8^+^ NKT cells^15,16^. The transcriptome of another subset is highly related to the cell adhesion and migration, unlike noncirculating and tissue-resident iNKT cells^52,53^. Therefore, we provided insight into the CD1d-independent NKT cells with single-cell resolution and showed two completely different transcriptional profiles of CD1d-independent NKT cell subsets.

The tissue-specific functions of iNKT cells have been thoroughly reviewed recently^28^. Functionally distinct subsets of iNKT cells, such as NKT1, NKT2, NKT17 and NKT10, were localized preferentially in lymphatic and nonlymphatic tissues^28^. Our work explored the subsets of NKT cells in different organs by using validated cell surface markers and found distinct distributions of subsets of NKT cells. In particular, Sca-1^−^CD62L^+^ NKT cells showed an increased frequency in the spleen, bone marrow, lymph nodes and lung compared with the liver. However, whether the transcriptome is altered in different locations remains to be elucidated. Future work needs to map NKT cells in different organs to provide a more accurate and comprehensive landscape for the study of tissue-specific functions.

As an inducer of IFN-γ secretion, IL-18 has been reported to augment the activity of both NK and T cells and has antitumor ability during tumor development^36–40^. In our work, we found a specific alteration pattern in the number of Sca-1^−^CD62L^+^ NKT cells during liver tumor development (Fig. 4f), which might indicate its function in tumor progression. We also found significantly higher levels of the components of the IL-18 receptor complex in Sca-1^−^CD62L^+^ NKT cells than in other NKT cell subsets. After administration of IL-12, IL18 and IL-2, Sca-1^−^CD62L^+^ CD1d-independent NKT cells showed a strong IFN-γ secretion ability, unlike the other NKT cell subsets. Treatment with IL-2 and IL-12 alone could upregulate the expression of the IL-18 receptor and enhance the secretion of IFN-γ. Previous work has demonstrated that IL-12/IL-18 NKT cells from the spleen adoptively transferred into syngeneic hosts could perform a strong antitumor function to inhibit the growth of ALC and MC57X syngeneic tumors^54^. The secretion of IL-2 and IFN-γ by NKT cells is important in these antitumor effects^54^. Besides, we found a specific higher expression of TCF1, a transcription factor that has been reported to be essential for the stem-like functions of tumor-infiltrating CD8^+^ T lymphocytes^55^, in Sca-1^−^CD62L^+^ CD1d-independent NKT cells than in other NKT cell subsets. Combined with our findings, we presumed that Sca-1^−^CD62L^+^ CD1d-independent NKT cells might affect the immune tumor microenvironment through the secretion of IFN-γ. These results revealed a NKT cell subset with potential for manipulation for cancer immunotherapy.

Given the importance of NKT cells in both the innate and adaptive immune systems, our work reclassified NKT cells with single-cell resolution and provided new insight into both CD1d-dependent and CD1d-independent NKT cells.

## Methods

### Animals

The C57BL/6 mice were purchased from Lingchang Biotech (Shanghai, China) as wild type control. The Jα18-deficient mice and CD1d-deficient mice, which have been describedL^56,57^, were originally obtained from Professor L. Bai (University of Science and Technology of China). Mice aged 8–10 weeks were used for experiments. All mice were maintained under pathogen-free conditions and genotyped by PCR before experiments. All animal experiments were conducted in compliance with National Institutes of Health guidelines and were approved by the institutional animal care and use committee of the Shanghai Institutes for Biological Sciences (Chinese Academy of Sciences).

### Antibodies and reagents

The fluorescently conjugated antibodies which were used for cell-surface staining and intracellular staining are listed in the Supplementary Table 1. CD1d-PBS57 (conjugated with PE) was obtained from the tetramer facility of the US National Institutes of Health as previously described^27^. Collagenase I used for tissue digestion was purchased from Gibco. Murine IL-2 and murine IL-12 p70 were obtained from PeproTech. Recombinant Mouse IL-18 was obtained from BioLegend.

### Tissue preparation and cell isolation

Tissue preparation and cell isolation were performed as previously described^27^. Briefly, the suspension of single cell from the thymus or lymph nodes was obtained by triturating and filtering through a nylon screen. Splenocytes were prepared by squeezing and filtering through a nylon screen, followed by red blood cell lysis before a second filtration. The livers or lungs were perfused with PBS before extraction to remove the blood cell. Then, the livers were minced and filtered through a cell strainer (40 μm; BD Biosciences). The lungs were minced into ~ 2 mm segments and incubated with shaking (200 rpm) at 37 °C for 50 min in RPMI medium (Gibco) containing 10% FBS (Sunrise), 70 U ml^−1^ collagenase I (Gibco). Leukocytes were isolated from the liver or digested lungs cell suspensions by density fractionation using discontinuous 40–70% (vol/vol) Percoll (GE Healthcare) gradients, followed by red blood cell lysis before filtration. Bone marrow cells were obtained by flushing the tibias and femurs, followed by red blood cell lysis before filtration.

### FACS analysis and cell sorting

For cell surface staining, cells were distributed in 5 ml polystyrene round-bottom tubes (BD Biosciences) and stained with the indicated antibodies for 40 min at 4 °C after Fc blocking (anti-CD16/CD32, BD Biosciences), followed by staining with viability dye (Fixable Viability Stain 450 or 780, BD Biosciences) before detection. For biotin conjugated antibody, PE streptavidin (BD Biosciences) were used before staining with viability dye. Intracellular staining for transcription factor (TCF-7) was performed by using a Foxp3 staining kit (eBioscience) according to the manufacturer’s protocol as previously described^27^. Cell fluorescence was performed on a four-laser BD LSRFortessa II and the data were analyzed with FlowJo software (TreeStar, Inc., Olten, Switzerland) as previously described^27^. Cell sorting was performed with a BD FACSAria II after cell surface staining as previously described^27^. The purity of sorted cell was greater than 95%.

### Quantitative real-time PCR analysis

Quantitative real-time PCR was performed as previously described^27^. Total RNA was extracted with TRIzol (Invitrogen) and reverse-transcribed by using the SuperScript III First-Strand Synthesis System (Invitrogen). The mRNA levels of the indicated genes were normalized to GAPDH by using real-time RT-PCR (QuantStudio6 Flex; Fujifilm) with a SYBR Green QPCR Master Mix (Toyobo). Primers used for qPCR were listed in the Supplementary Table 2.

### In vitro cell stimulation and cell enrichment

For the production of IFN-γ in vitro, sorted cells were incubated with indicated cytokines in a Nunc MicroWell 96-Well, Nunclon Delta-Treated, U-Shaped-Bottom Microplate (Thermo Fisher Scientific) for the indicated times and then cell supernatants were harvested for Elisa. To enrich Sca1^−^CD62L^+^ NKT cells from the spleen for cell stimulation, cell suspensions were depleted of B220^+^ (RA3-6B2) cells before sorting using biotinylated antibodies (BioLegend, 103204) bound to Dynabeads biotin binder (Invitrogen).

### Elisa

The cytokines in cell supernatant were assessed by using a mouse IFN-γ ELISA kit (R&D Systems) according to the manufacturer’s instructions.

### Hydrodynamic tail vein injection

The hydrodynamic injection was performed as previously described^35^. Briefly, 10 μg of the plasmids encoding myr-AKT1 and N-RasV12 along with 1.8 μg sleeping beauty transposase were diluted in 2 mL saline (0.9% NaCl), filtered through a 0.22-μm filter and injected in to a lateral tail vein of 6 to 8 weeks WT mice in 5 to 7 s. Mice were sacrificed at the indicated time.

### Single-cell RNA-seq library construction

First, individual cells were manually picked, lysed and subjected to first-strand cDNA synthesis as reported^58,59^. Then, second-strand cDNA was synthesized, amplified and fragmented. RNA-seq libraries were prepared according to the instruction manual of the KAPA Hyper Prep Kit (KAPA Biosystems). Finally, the libraries were checked, pooled together and sequenced with paired-end 150-bp reads on an Illumina HiSeq X-Ten platform.

### Processing the single-cell RNA-seq data.

Raw reads were first trimmed by Cutadapt (v1.15) and clean reads for each individual cell were classified according to cell barcodes on Read#2. Then, Read#1 was aligned to the mm9 mouse transcriptome by Tophat (v2.0.12). After de-duplication based on UMIs, the transcript copy numbers of each gene were counted by HTseq (v0.10.0). Cells with more than 500 detected genes, and transcripts between 10,000 and 600,000 were retained. Then, cells with high ratio of ERCC spike-ins were further removed by the “isOutlier” function in the R package “scater”. The R package “Seurat” was adopted to perform the downstream analysis including identification of differentially expressed genes (DEGs, |ln(fold change)|> 0.25 and adjust.p < 0.05 were considered significant) and calculation of similarity scores.

### Quantification and statistical analysis

The experiments described above were performed three or more times. Data were expressed as mean ± sd. The two-tailed unpaired Student’s t-test was used to determine statistical significance unless otherwise indicated as previously described^60^. For all experiments: *P < 0.05; **P < 0.001; ***P < 0.0001, ****P < 0.0001 by Student’s *t*-test; N.S.: no significance.

## Supplementary information

Supplementary Information.

## Data Availability

The data supporting this study are available in the article and its supplemental information files or from the corresponding author upon request. Single cell RNA sequencing data that support the results of this study were deposited in the NCBI Gene Expression Omnibus (GEO) under the accession number GSE152962, the token is: cvarscwspjwjlcn.
