# Supplementary material for: Unveiling the heterogeneity of NKT cells in the liver through single cell RNA sequencing
Source: Sci Rep. 2020 Nov 10;10:19453. doi: 10.1038/s41598-020-76659-1 (PMC7655820; doi:10.1038/s41598-020-76659-1)
Supplement: Supplementary file 1 — Supplementary Information. [file 41598_2020_76659_MOESM1_ESM.pdf]

Unveiling the heterogeneity of NKT cells in the liver through single cell RNA sequencing

Hao Shen[a,1], Chan Gu[d,1], Tao Liang[a], Haifeng Liu[a], Fan Guo[d,e,*] and Xiaolong Liu[a,b,c*]

[a]State Key Laboratory of Cell Biology, CAS Center for Excellence in Molecular Cell Science, Shanghai Institute of Biochemistry and Cell Biology, Chinese Academy of Sciences, University of Chinese Academy of Sciences, China

[b]School of Life Sciences, Hangzhou Institute for Advanced Study, UCAS, Hangzhou 310024, China.

[c]School of Life Science and Technology, ShanghaiTech University, Shanghai, 200031, China

[d]Center for Translational Medicine, Ministry of Education Key Laboratory of Birth Defects and Related Diseases of Women and Children, Department of Obstetrics and Gynecology, West China Second Hospital, Sichuan University, Chengdu, Sichuan 610041, China

[e]Ministry of Education Key Laboratory of Bio-resource and Eco-environment,

College of Life Sciences, Sichuan University, Chengdu, Sichuan 610041, China

[1]These authors contributed equally to this work

*Correspondence and requests for materials should be addressed to X.L.

(liux@sibs.ac.cn) and F.G. (guofan@scu.edu.cn)

Phone: 86-21-54921176

Fax: 86-21-54921178

Supplementary Information

Supplementary Fig. 1 Quality of scRNA-seq data.

Number of detected UMIs and genes in individual cells.

Supplementary Fig. 2 Gene expression across all clusters and validation of the differential genes at protein level.

a Violin plots showing the expression of *Klra9* and *Klra7* across all clusters. **b** Representative histograms of the expression of ICOS, CD5, CD127, TCF1/TCF7 and CD244 in NKT cell subsets in the liver from WT mice by flow cytometry. C0 enriched: *Icos* (ICOS) and *Cd5* (CD5); C1 enriched: *Cd244* (CD244); C2 enriched: *Il7r* (CD127) and *Tcf7* (TCF1/TCF7). Sca-1^{+}CD62L^{-} NKT cells represent C0, Sca-1^{-}CD62L^{-} NKT cells represent C1 and Sca-1^{-}CD62L^{+} NKT cells represent C2.

For **a**, gene expression in each cluster was calculated from the combination of all liver samples from WT, Jα18-deficient and CD1d-deficient mice, unless otherwise indicated. For **b**, the data are representative of or combined from at least three independent experiments, unless otherwise indicated.

Supplementary Fig. 3 Organ distribution of NKT cell subsets.

a Flow cytometry analysis of NK1.1 and CD3e expression in the thymus, spleen, lymph nodes, bone marrow, liver, lung and blood; Flow cytometry analysis of Sca-1 and CD62L expression in ($NK1.1^{+}CD3e^{+}$) NKT cells in the thymus, spleen, lymph nodes, bone marrow, liver, lung and blood; **b** The total number of $Sca\text{-}1^{+}CD62L^{-}$, $Sca\text{-}1^{-}CD62L^{-}$ and $Sca\text{-}1^{-}CD62L^{+}$ NKT cells in the liver and spleen was quantified in WT mice. (n=5); **c** Flow cytometry analysis of $CD3e^{int}CD1d\text{-}PBS57^{+}$ cells in the liver; Flow cytometry analysis of NK1.1 and CD3e expression of the $CD3e^{int}CD1d\text{-}PBS57^{+}$ cells; **d** The frequency of $NK1.1^{-}$ $CD3e^{int}CD1d\text{-}PBS57^{+}$ cells in $CD3e^{int}CD1d\text{-}PBS57^{+}$ cells. (n=6).

The data are presented as the mean ± s.d. For all panels: $^{**}P < 0.01$; $^{****}P < 0.0001$ by Student's t-test; N.S.: no significance. All data are representative of or combined from at least three independent experiments, unless otherwise indicated.

Supplementary Table 1. Primers used for qPCR.

Supplementary Table 2. Antibodies used for FACS analysis.

Supplementary Fig. 1

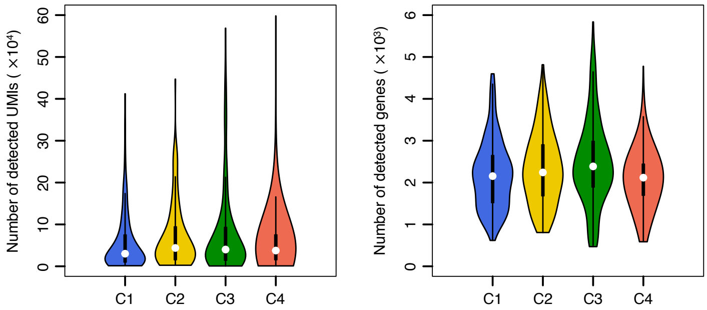

Supplementary Fig. 2

a

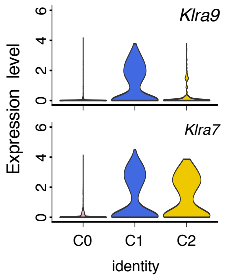

b

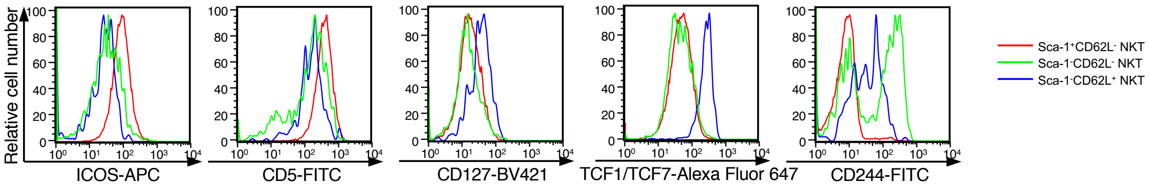

Supplementary Fig. 3

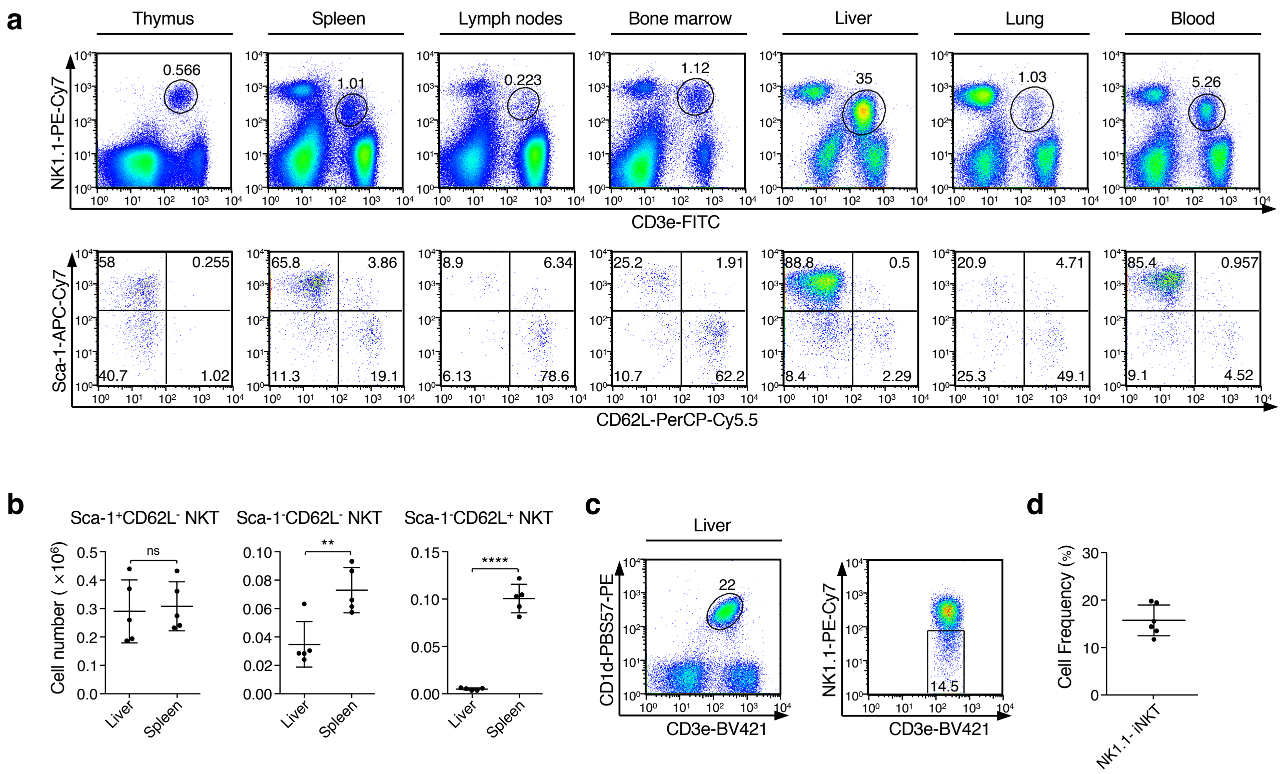

Supplementary Table 1

Primer name	Sequence	Application
Ly6a-forward	5′ -AGGAGGCAGCAGTTATTGTGG-3′	qPCR
Ly6a-reverse	5′ -CGTTGACCTTAGTACCCAGGA-3′	qPCR
Il4-forward	5′ -GGTCTCAACCCCCAGCTAGT-3′	qPCR
Il4-reverse	5′ -GCCGATGATCTCTCTCAAGTGAT-3′	qPCR
Lyst-forward	5′ -AGCAGAAGGTGATAGACCAGAA-3′	qPCR
Lyst-reverse	5′ -CCCACACTTGGATCATCAATGC-3′	qPCR
Rora-forward	5′ -GTGGAGACAAATCGTCAGGAAT-3′	qPCR
Rora-reverse	5′ -TGGTCCGATCAATCAAACAGTTC-3′	qPCR
Cd6-forward	5′ -GGAGGGCTACTGCAATGATCC-3′	qPCR
Cd6-reverse	5′ -GTGAGGGGACTCTTCTCAGAAT-3′	qPCR
Sell-forward	5′ -TGACGCCTGTCACAAACGA-3′	qPCR
Sell-reverse	5′ -GGCTGGCAAGAGGCTGTGT-3′	qPCR
Pdlim1-forward	5′ -TCGATGGGGAAGATACCAGCA-3′	qPCR
Pdlim1-reverse	5′ -TCTGTTCAGACCTGGATACTGTG-3′	qPCR
Tcf7-forward	5′ -AGCTTTCTCCACTCTACGAACA-3′	qPCR
Tcf7-reverse	5′ -AATCCAGAGAGATCGGGGGTC-3′	qPCR
S1pr1-forward	5′ -ATGGTGTCCACTAGCATCCC-3′	qPCR
S1pr1-reverse	5′ -CGATGTTCAACTTGCCTGTGTAG-3′	qPCR
Emb-forward	5′ -TGAGGGCGATCCCACAGAT-3′	qPCR
Emb-reverse	5′ -CCGTCACTGAGATATTACAGCTC-3′	qPCR
Cd8a-forward	5′ -CCGTTGACCCGCTTTCTGT-3′	qPCR
Cd8a-reverse	5′ -CGGCGTCCATTTTCTTTGGAA-3′	qPCR
Ccl4-forward	5′ -TTCCTGCTGTTTCTCTTACACCT-3′	qPCR
Ccl4-reverse	5′ -CTGTCTGCCTCTTTTGGTCAG-3′	qPCR
Klra5-forward	5′ -CCAGAGGTCACTTACTCAACT-3′	qPCR
Klra5-reverse	5′ -ATTCCAAGCGATCTCACAGT-3′	qPCR
Cd244-forward	5′ -GAATATGTCAAGGACTCACGAGC-3′	qPCR
Cd244-reverse	5′ -GTCCAATTCTCTTTGTCCCCTC-3′	qPCR
Itgax-forward	5′ -CTGGATAGCCTTTCTTCTGCTG-3′	qPCR
Itgax-reverse	5′ -GCACACTGTGTCCGAACTCA-3′	qPCR
Gapdh-forward	5′ -ACTCCACTCACGGCAAATTCA-3′	qPCR
Gapdh-reverse	5′ -GCCTCACCCCATTTGATGTT-3′	qPCR

Supplementary Table 2

Antibodies	Source	Identifier
Armenian Hamster monoclonal anti-CD3e FITC conjugated (clone: 145-2C11)	BD Biosciences	Cat# 553062
Rat monoclonal anti-CD62L PerCP/Cy5.5 conjugated (clone: MEL-14)	BioLegend	Cat# 104432
Mouse monoclonal anti-NK-1.1 PE/Cy7 conjugated (clone: PK136)	BioLegend	Cat# 108714
Rat monoclonal anti-Ly-6A/E (Sca-1) APC/Cy7 conjugated (clone: D7)	BioLegend	Cat# 108126
Rat monoclonal anti-CD45R/B220 Biotin conjugated (RA3-6B2)	BioLegend	Cat# 103204
Armenian Hamster monoclonal anti-CD278 (ICOS) APC conjugated (clone: C398.4A)	BioLegend	Cat# 313509
Rat monoclonal anti-CD218a (IL-18Rα) Alexa Flour 647 conjugated (clone: BG/IL18RA)	BioLegend	Cat# 132903
Rat monoclonal anti-CD127 (IL-7Rα) BV421 conjugated (clone: A7R34)	BioLegend	Cat# 135023
Rat monoclonal anti-CD5 FITC conjugated (clone: 53-7.3)	BioLegend	Cat# 100605
Mouse monoclonal anti-CD244.2 (2B4 B6 Alloantigen) FITC conjugated (clone: m2B4(B6)458.1)	BioLegend	Cat# 133503
Rabbit monoclonal anti-TCF1/TCF7 Alexa Flour 647 conjugated (clone: C63D9)	Cell Signaling Technology	Cat# 6709S
Rat monoclonal anti-Ly-6A/E PE conjugated (clone: D7)	BD Biosciences	Cat# 553108
Mouse monoclonal anti-CD212 Biotin conjugated (114)	BD Biosciences	Cat# 551973
Armenian Hamster monoclonal anti-CD3e BV421 conjugated (clone: 145-2C11)	BD Biosciences	Cat# 562600
Rat monoclonal anti-CD8a APC conjugated (clone: 53-6.7)	Thermo Fisher Scientific	Cat# 17-0081-83
Rat monoclonal anti CD16/CD32 (Mouse BD Fc Block) unconjugated (clone: 2.4G2)	BD Biosciences	Cat# 553142
